# Endoscopic Diagnosis of Chronic Atrophic Gastritis and Early Gastric Cancer: From Basics to Advanced Imaging

**DOI:** 10.3390/cancers18111846

**Published:** 2026-06-04

**Authors:** Matthew Banks, David Graham

**Affiliations:** Department of Gastroenterology, University College Hospital, London NW1 2PG, UK; david.graham14@nhs.net

**Keywords:** endoscopy, gastric cancer, gastroscopy, imaging, artificial intelligence, gastrointestinal endoscopy, endoscopic, pathology

## Abstract

Chronic atrophic gastritis (CAG) is the principal precursor lesion for gastric adenocarcinoma and represents a key target for endoscopic surveillance and early intervention. Although the global age-standardised incidence of gastric cancer has declined over recent decades, the absolute number of cases continues to rise because of population ageing and increasing incidence in younger individuals. The prognosis remains poor in advanced disease, whereas early gastric cancer (EGC) detected at a mucosal stage is associated with excellent long-term survival and may be curable with endoscopic resection. Consequently, high-quality endoscopic detection of premalignant gastric lesions is essential to reduce gastric cancer mortality. This review summarises current concepts in the endoscopic diagnosis of CAG, gastric intestinal metaplasia (GIM), and EGC, from conventional white-light endoscopy through to advanced imaging and artificial intelligence (AI)-assisted systems. Fundamental principles of high-quality oesophagogastroduodenoscopy are discussed, including adequate inspection time, systematic mucosal assessment, mucosal cleansing, and standardised photo-documentation. Characteristic endoscopic appearances of normal gastric mucosa, atrophy, and intestinal metaplasia are reviewed, alongside established staging systems including the Kimura–Takemoto and EGGIM classifications. The role of image-enhanced endoscopy is examined in detail, including narrow-band imaging, linked colour imaging, texture and colour enhancement imaging, and magnification optical enhancement. These modalities improve visualisation of pit patterns, microvascular architecture, and hallmark features of intestinal metaplasia such as the light blue crest sign, substantially increasing diagnostic sensitivity and specificity compared with conventional white light imaging alone. Advanced imaging combined with magnification also enhances the detection and characterisation of EGC. Emerging evidence regarding AI-assisted endoscopy demonstrates promising diagnostic accuracy for CAG, GIM, and early neoplasia, with improved lesion detection and reduced miss rates in several studies. However, limitations relating to external validation, generalisability, and integration into routine practice remain. Continued advances in optical imaging, structured training, and AI-supported diagnostics are likely to play an increasingly important role in improving early gastric cancer detection and surveillance outcomes worldwide.

## Simple Summary

Chronic atrophic gastritis (CAG) is the main precursor lesion for gastric adenocarcinoma and an important target for endoscopic surveillance and early intervention. Although age-standardised gastric cancer rates have declined globally, total case numbers continue to rise due to ageing populations and an increasing incidence in younger individuals. Early gastric cancer detected at a mucosal stage carries an excellent prognosis and may be curable endoscopically, emphasising the importance of accurate endoscopic diagnosis. This review outlines current approaches to detecting CAG, gastric intestinal metaplasia (GIM), and early gastric cancer using both conventional and advanced endoscopic techniques. Key principles of high-quality oesophagogastroduodenoscopy include careful mucosal inspection, adequate examination time, mucosal cleansing, and standardised documentation. Advanced imaging modalities such as narrow-band imaging and linked colour imaging improve visualisation of mucosal and vascular patterns, thereby increasing diagnostic accuracy. Artificial intelligence-assisted endoscopy also shows promise in improving lesion detection and reducing miss rates, although further validation is required before widespread routine implementation.

## 1. Introduction

The most common precursor for gastric cancer is chronic atrophic gastritis (CAG). The endoscopic diagnosis of CAG and the subsequent detection and treatment of neoplastic lesions through surveillance remain key interventions in order to reduce the burden of advanced gastric cancer.

Gastric cancer remains a significant worldwide problem, and ranks as the fifth highest for incidence and fourth for mortality globally. The overall global trend has shown that there is a paradoxical pattern, with age-standardised gastric cancer incidence declining, whereas the absolute number of cases is increasing most likely due to population growth and an increasing age of the population. More recently it has been shown that early-onset gastric cancer in those <50 has been increasing, a trend also seen in colorectal cancer incidence [1,2,3]. The global age-standardised incidence rate (ASIR) decreased from 35.3 per 100,000 in 1990 to 20.2 per 100,000 in 2021, with an average annual percentage change of approximately −2.1% [4,5].

This decline is attributed to a reduced *Helicobacter pylori* prevalence, and possibly improved food preservation and refrigeration, decreased smoking, and dietary improvements [4]. East Asian countries have experienced the most dramatic reductions: South Korea’s ASIR dropped from 71.2 to 25.8 per 100,000 (EAPC −3.54%), and Japan’s from 64.1 to 25.5 per 100,000 (EAPC −2.98%) between 1990 and 2021. Despite these declining rates, the total number of new cases globally increased from approximately 800,000 to 1.23 million over the same period, driven by population growth and ageing [5]. It has been hypothesised that the increase in those under 50 years of age may be accounted for by a rising prevalence of autoimmune chronic gastritis and dysbiosis of the gastric microbiome [6].

Asia bears the greatest burden, accounting for approximately 75% of all new cases and deaths. Incident cases in Asia are projected to increase by 72.2% by 2040 due to population ageing, despite declining age-standardised rates [7].

The late diagnosis of gastric cancer contributes to these poor outcomes, with a UK ten-year survival 15.5% for men and 19.5% for women [8]

Early detection and prompt treatment of gastric cancer are crucial for improving clinical outcomes. For instance, early gastric cancer (EGC) carries a favourable prognosis, with five-year survival rates ranging from 69% to 82%. When the disease is limited to the mucosa (stage T1a), endoscopic resection (ER) is often curative and is associated with excellent outcomes, with five-year survival rates reaching up to 92% [9,10].

Currently, endoscopy is the most effective test for the diagnosis of early gastric cancers and pre-malignant conditions. The procedure, otherwise known as oesophago-gastroduodenoscopy or OGD is safe, well-tolerated and can be performed both awake or with sedation. Advanced cancers are easily evident and detectable during endoscopy, however early disease or the precursors to cancer can be more subtle, requiring extensive training and the use of enhanced imaging modalities. The vast majority of gastric adenocarcinomas develop on a background of chronic atrophic gastritis (CAG), with an increase in the risk of cancer dependent upon the extent of atrophy and gastric intestinal metaplasia. The increased risk of cancer is the basis for surveillance in these patients. There are now a number of international and national guidelines that have been developed to create a framework for the surveillance of those at risk of gastric cancer [11,12,13,14].

A recent review emphasises both primary and secondary prevention strategies in gastric cancer. Early detection and eradication of *Helicobacter pylori* infection represent primary prevention, while identification of early gastric cancer (EGC) suitable for endoscopic resection serves as secondary prevention to reduce mortality. The paper highlights the Kyoto Classification of Gastritis as an important endoscopic scoring system for gastric cancer risk stratification. High scores for gastric atrophy and intestinal metaplasia are strongly associated with the risk of differentiated gastric cancer risk. In contrast, undifferentiated gastric cancer is linked to enlarged gastric folds and nodular gastritis, particularly in younger patients. Enlarged folds are also associated with deeper submucosal invasion. Diffuse redness correlates with a progressive increase in the annual gastric cancer incidence according to severity. Although the eradication of *H. pylori* reduces the recurrence risk by approximately one-third, residual cancer risk persists when premalignant mucosal changes have already developed prior to eradication [15]. A landmark publication, Screening and eradication of Helicobacter pylori for gastric cancer prevention: Taipei Global Consensus II provides 28 evidence-based recommendations supporting population-based screening and eradication strategies for *Helicobacter pylori* infection. The consensus establishes that eradication significantly reduces gastric cancer incidence and mortality across all adult age groups, with the greatest benefit achieved before the development of atrophic gastritis or intestinal metaplasia. The document also supports the use of urea breath testing and monoclonal stool antigen testing as preferred non-invasive screening modalities and highlights the potential value of family-based eradication programmes in reducing reinfection and household transmission [16].

This review will explore the current guidance as well as the latest advances in optical imaging for the detection and surveillance of chronic atrophic gastritis and early gastric cancer. We will also focus on the emerging endoscopic technologies including artificial intelligence. Although the paper is not a systematic literature review, a thorough search of the world literature was undertaken specifically for English-language publications using the following key words: Chronic atrophic gastritis, Helicobacter pylori, gastric cancer, endoscopy, enhanced endoscopic imaging, AI, optical diagnosis, gastritis. We included high-quality studies depending on their relevance, including meta-analyses, guidelines and systematic reviews from 1980 onwards. The Cochrane database, Pubmed and Embase were used as the principal literature databases and the LeanLibrary Workspace was utilised to compile the included publications. The only exclusion criterion was articles not written in English.

## 2. Chronic Atrophic Gastritis: Background

The histological definition of CAG requires the presence of chronic inflammation in addition to the loss of gastric glands. Following the progression of the condition, the native gastric glands are replaced by metaplastic glands to produce gastric intestinal metaplasia (GIM). The precursor to atrophy is inflammation, induced most commonly by *Helicobacter pylori*, followed by autoimmune gastritis. The Correa group described a stepwise transformation from *Helicobacter pylori*-induced chronic inflammation to CAG, GIM, dysplasia and finally cancer [17].

## 3. Performing High Quality Endoscopy: Basics

There are a number of fundamental elements required to perform a high-quality endoscopy before utilising more advanced techniques of imaging. The principles include both technical and non-technical skills, the latter including leadership, situational awareness, and adequate communications skills with the patient and other members of staff. Preceding the procedure, it is important to verify that all administrative and informatics processes are in place to ensure adequate patient preparation, provision of information regarding the procedure, appropriate timing and the management and communication of results. There is published guidance on high-quality endoscopy with key performance indicators including a sufficient time (>7 min), the use of mucosal cleansing agents, and sufficient photo-documentation of all anatomical landmarks and abnormalities, leading to adequate mucosal visualisation [18,19].

The European Society of Gastrointestinal Endoscopy (ESGE) guidelines for OGD have recommended that the inspection time for the procedure should be at least 7 min from intubation to extubation [19]. This recommendation was based on a study investigating the accuracy of detecting early neoplastic lesions in the stomach. In a retrospective cohort study, the authors demonstrated that a ‘slow’ endoscopist (>7 min examination) detected 2.4 times (OR) more high-risk gastric lesions (Atrophy and GIM) and 3.4 (OR) times more dysplasia and cancer compared with a ‘fast’ endoscopist (<7 min examination) [20,21].

Failure to meet the proposed quality standards will increase the likelihood of a post-endoscopy upper GI cancers (PEUGIC), which are defined as a cancer diagnosis occurring between 3 months and 3 years after a previously negative endoscopy. Cancer mis-rates up to 3 years after a normal endoscopy were as high as 11.3% in a meta-analysis from 2014 [22]. Furthermore, variation in quality was highlighted by a Japanese study that found that detection rates of Early gastric cancer (EGC) varied by endoscopist from 0.09 to 2.87%. In addition, endoscopists with higher EGC detection rates were better at detecting minute EGCs (<5 mm) [23].

Although there is limited data on the quality of a complete endoscopic procedure, in that no clinical trials have directly clarified key performance measures for improving gastric cancer detection, there is some emerging data. In the UK, a large study of 9068 PEUGIC cases identified from National Cancer Registration and Hospital Episode Statistics datasets, found that the national surveillance PEUGIC rate (For CAG and Barrett’s oesophagus) was 21.1% SD ± 5.4 varying from 7.5% to 37.0% between providers. The ‘failed’ surveillance PEUGIC rate (stage 2 cancer or greater) was 13.7% SD ± 4.7 varying from 7.5% to 37%, confirming a wide variation in performance. It was suggested that the adjusted provider PEUGIC rate minus successful surveillance (stage 1 at diagnosis) is the most appropriate key performance indicator utilized to identify poor performers, aiming to provide upskilling and support [24].

Furthermore, factors associated with a higher PEUGIC rate were investigated in a large national UK study involving 144 sites, whereby each case was reviewed via an online root cause analysis algorithm. The cases included both diagnostic and surveillance. Interestingly, among the surveillance cases, a number of PEUGICs were found to be outside of the surveillance area, a phenomenon termed a ‘fixation error’. In essence factors associated with a higher PEUGIC related to non adherence to best practice, and included limited time slots for the procedure, procedure undertaken outside of a centre of excellence and delays in recall for the surveillance [25].

There is low-quality data that suggests photographic documentation might be an indirect quality indicator. Studies have demonstrated that longer procedure times resulted in more image capture and a higher pathology detection rate [21]. In addition, different societies have recommended that specific areas in the stomach should be photographically documented, including the cardia and fundus in inversion, the corpus in forward view including the lesser curvature, the corpus in retroflex view including the greater curvature, the angulus in partial inversion, and the antrum [11,18,19].

**Figure 1:**
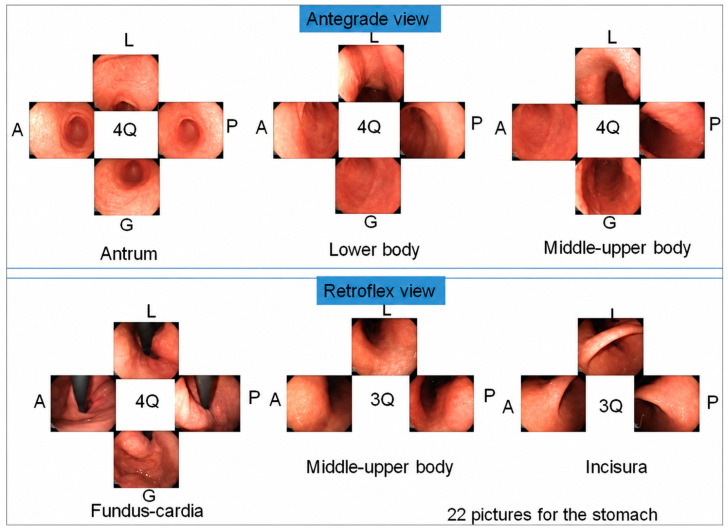
Systematic screening protocol for the stomach (SSS): this structured, station-based approach involves sequential inspection and photographic documentation of each anatomical region of the stomach, performed in a clockwise or counterclockwise sequence. A total of 22 standardised images are obtained and organised in accordance with the procedural order to ensure comprehensive mucosal assessment. Abbreviations: Q—quadrant; L—lesser curvature; A—anterior wall; G—greater curvature; P—posterior wall.

An endoscopic systematic screening protocol for CAG has been developed in Japan, and revised by the Japanese Society of Gastroenterological Cancer Screening to a simplified, version, proposing a minimum quality standard described as a ‘systematic screening protocol for the stomach’ [26,27,28,29]. This station-based system recommends that each area of the stomach be viewed and photographed in either a clockwise or counterclockwise manner. There are a total of 22 images, which are arranged according to the order of the procedure. Additional pictures are taken of lesions (Figure 1). If a similar systematic protocol with 20 images is incorporated into training, Zhang et al. demonstrated that the detection rate of early gastric cancer increases from 0.2% to 2.3% [29].

## 4. Advanced Imaging: Detection of Gastric Atrophy and Intestinal Metaplasia

In order to detect abnormalities in the stomach, it is important to understand the normal appearances of the mucosa under both WLE and with enhanced imaging.

### 4.1. Normal Gastric Appearances: White Light Endoscopy

**Figure 2:**
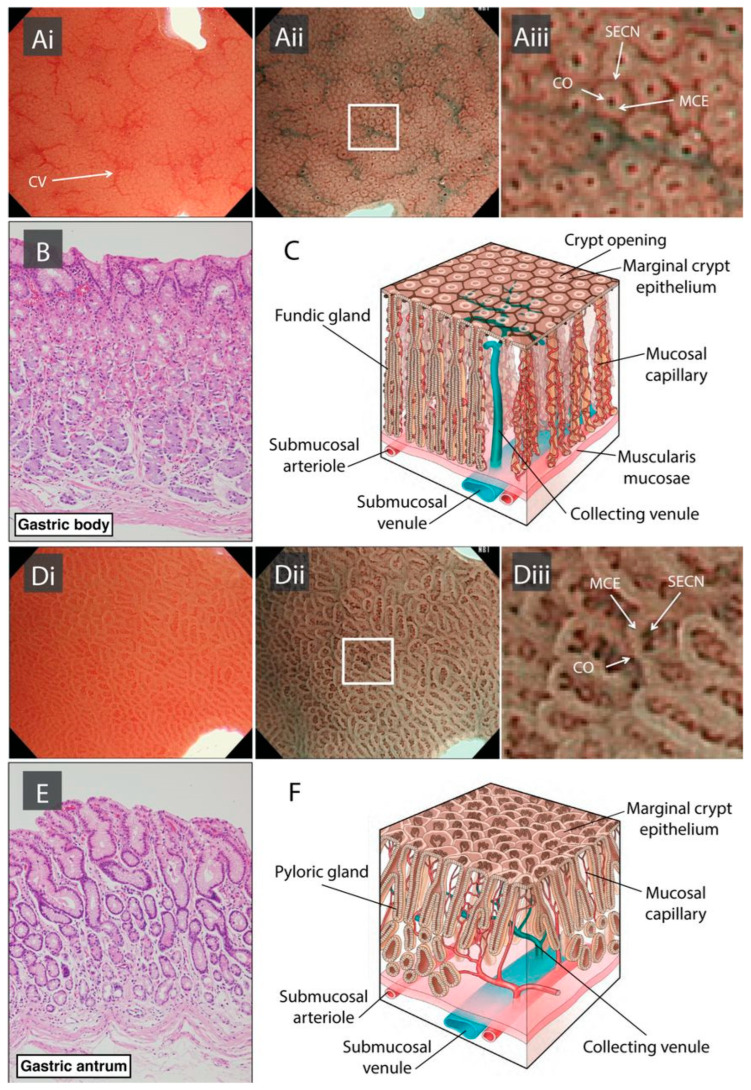
Normal gastric corpus and antral mucosa surface architecture on white light, enhanced imaging, and magnification endoscopy. The characteristic round pit pattern of the corpus (**Ai**) and the elongated pit pattern of the antrum (**Di**) are visible even without image enhancement or magnification. Within the corpus (**Ai**), prominent red collecting venules (CV) and round, dark-red crypt openings (CO) are identified. With narrow-band imaging (NBI), the vascular structures become more clearly defined (**Aii**,**Dii**). Magnification NBI (Enlarged box in **Aii**,**Dii**) demonstrates several distinct anatomical features, including dark-brown crypt openings (CO), the dark-brown subepithelial capillary network (SECN), and the light-brown marginal crypt epithelium (MCE). In the corpus mucosa, the pattern consists of dark, rounded crypt openings encircled by the paler marginal crypt epithelium, which is itself bordered by the darker circular SECN (**Aii**,**Aiii**). By comparison, the antral mucosa displays dark, obliquely orientated crypt openings with a grooved configuration. Here, the lighter ridged or villiform marginal crypt epithelium surrounds the darker SECN, producing the characteristic “groove-type” pattern (**Dii**,**Diii**). Corresponding histopathological appearances are demonstrated for the corpus in panels (**B**,**C**), and for the antrum in panels (**E**,**F**).

The normal gastric body or corpus displays folds called rugae, which are absent in the antrum and fundus. The colour of the normal gastric mucosa is a dark rose or red with a regular arrangement of the collecting venules or RACs. The presence of RACs is characteristic of a normal stomach without *H. pylori* (sensitivity 93%, specificity 48%) [30,31]. The round pit patterns of the gastric body and the oblique pit patterns of the antrum can be seen with high-resolution WLE (Figure 2(Di)).

### 4.2. Normal Gastric Appearances: Magnification and Enhanced Endoscopy

The two principal features that define the gastric mucosa on magnifying endoscopy are the surface (epithelial) microstructure and the microvascular architecture.

In the gastric corpus, the mucosa is composed of relatively straight, tubular glands that open onto the surface via round crypt (foveolar) openings. Endoscopically, these appear as dark, round pits, which are encircled by the marginal crypt epithelium (MCE)—a paler, whitish rim. Surrounding this epithelial component is the subepithelial capillary network (SECN), which is visualised as a darker, regular, honeycomb-like vascular pattern.

This arrangement produces the characteristic foveolar-type pattern, in which light areas (glandular epithelium) are surrounded by dark areas (capillaries). These endoscopic findings correlate closely with the underlying histological architecture of tightly packed, vertically oriented tubular glands within the corpus mucosa.

On narrow-band imaging (NBI), this is typically described as a pattern of regular microvasculature with circular mucosal structures, which has been shown to correlate with normal histology with an accuracy of approximately 83% (95% CI 75–90%) [32].

In contrast, the gastric antrum demonstrates a distinctly different architecture. The glands are branched, tortuous, and obliquely oriented, rather than straight. The surface openings appear as elongated or grooved pits, rather than round pits. The marginal crypt epithelium forms a ridgeed or villiform pattern, appearing as lighter, elevated structures.

The subepithelial capillary network in the antrum is correspondingly different, presenting as coiled or wavy vessels that run between the epithelial ridges. This combination of grooved pit openings, villiform epithelial ridges, and coiled microvasculature gives rise to the so-called groove-type pattern on magnifying endoscopy.

These endoscopic features reflect the underlying papillary and branched glandular architecture of the antral mucosa on histology, distinguishing it clearly from the tubular gland pattern seen in the corpus.

### 4.3. Endoscopic Appearances of Gastric Atrophy

There are four key features that enable the endoscopist to detect atrophy, including the mucosal pallor, the loss of gastric rugae, and the enhanced visibility of blood vessels which were first described by Nakayama et al., Uedo et al. and Yao et al. [33,34,35]. The identification and positioning of the atrophic border, the latter being the demarcation between the pale atrophic mucosa and the normal mucosa of the gastric corpus, constitute a fifth identifier. The atrophic border defines the extent of disease and progresses from the distal to proximal stomach, and from the lesser to the greater curve, following the colonisation of the mucosa with *Helicobacter pylori* (the correa cascade). High-definition white-light endoscopy (HD-WLE) offers significantly improved sensitivity for identifying atrophy and GIM when compared to conventional white-light endoscopy.

Long-term cohort data indicate that the Kimura–Takemoto classification serves as a valuable endoscopic risk stratification tool for predicting the development of gastric adenocarcinoma [36]. This classification system is based on identifying and mapping the atrophic border. The position and proximal extension of this border are used to determine the extent and severity of gastric atrophy (GA).

Evidence from cross-sectional, cluster-sampled comparative studies between populations in the United Kingdom and Japan has demonstrated that endoscopic grading using this system correlates closely with histopathological assessment. In particular, it has shown good diagnostic performance in identifying histological atrophy, with a low rate of false-negative findings [37,38]. However, further validation in larger and more diverse non-Japanese populations is required to confirm its generalisability.

**Figure 3:**
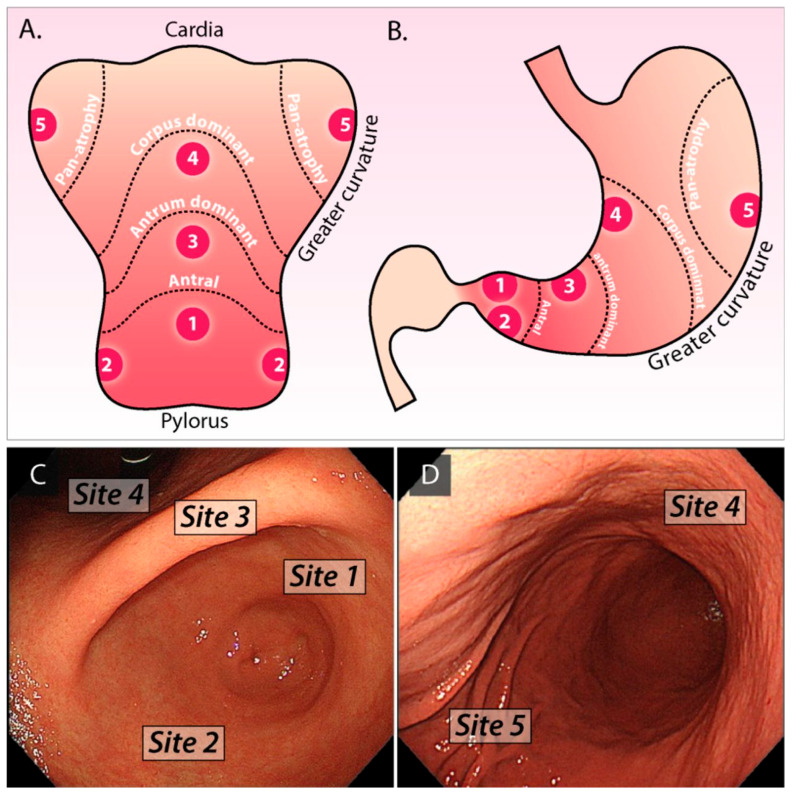
The Integrated and Modified Kimura and Sydney Biopsy System. The modified Kimura staging system divides the extent of atrophy into antrum only (antral), antrum to incisura (antral dominant), antrum to lesser curve (corpus dominant), and antrum, lesser curve and greater curve (pan-atrophy). This system integrates Sydney protocol biopsies which should be taken from the antrum (site 1 and 2), incisura (site 3), lesser curve (site 4) and greater curve (site 5). The anatomical CAG boundaries and biopsy sites can be seen in the splayed (**A**) and cross-sectional cartoon (**B**) of the stomach. The biopsy sites defined in the endoscopic retroflexed (**C**) and forward view (**D**).

To improve clinical applicability, the Kimura–Takemoto classification has been adapted into a simplified staging framework based on the anatomical distribution of atrophy (Figure 3). This modified system categorises disease extent as:

- Antral-limited atrophy (confined to the antrum)
- Antral-predominant atrophy (extending from the antrum to the incisura)
- Corpus-predominant atrophy (extending along the lesser curvature into the corpus) and
- Pan-atrophy (involving the antrum, as well as both the lesser and greater curvatures of the corpus)

This simplified staging approach is designed to integrate with the Sydney biopsy protocol, thereby aligning endoscopic visual assessment with systematic histological sampling to provide a more comprehensive evaluation of gastric atrophy.

In essence, the Kimura–Takemoto classification is used to visually assess the extent of atrophy and guide targeted biopsies (Figure 3).

### 4.4. Endoscopic Appearances of Gastric Intestinal Metaplasia

On white-light endoscopy, gastric intestinal metaplasia (GIM) typically manifests as small, grey-white, slightly elevated plaques, often set against a background of heterogeneous mucosa comprising patchy erythematous and pale, atrophic areas. This results in an overall appearance of an irregular, uneven mucosal surface. GIM can be patchy or diffuse, depending on its extent. When patchy, individual islands are evident as pale plaques, either flat or slightly elevated on white-light endoscopy (WLE), usually surrounded by atrophic mucosa. When GIM is diffuse, the entire mucosa appears uneven and nodular.

Despite these recognised features, the diagnostic accuracy of standard white-light endoscopy alone, particularly without high-definition imaging or image-enhanced modalities, is limited and unreliable for the detection of GIM.

As areas of gastric intestinal metaplasia develop, the normal straight, tubular glands of the gastric corpus undergo architectural distortion, becoming elongated and reorganised into a groove-type pattern, resembling the antral mucosa (otherwise known as antralisation of the corpus), or adopting a more villiform configuration akin to the intestinal epithelium.

In the gastric corpus, these metaplastic changes are typically readily distinguishable from the surrounding normal mucosa, both on high-resolution white-light endoscopy and with image-enhanced techniques. In contrast, GIM within the antrum is more challenging to identify, as the native antral mucosal architecture is already oblique, grooved, and structurally similar, making differentiation less straightforward (Figure 2).

Additional characteristic endoscopic features of GIM include the light blue crest (LBC), the marginal turbid band (MTB), and the presence of a white opaque substance representing lipid droplets within the epithelium, which can obscure the underlying subepithelial capillary network.

The light blue crest is observed on narrow-band imaging (NBI) as a fine blue-white line along the epithelial crest of the mucosal surface and is considered a highly specific marker for GIM.

The marginal turbid band appears as a whitish, turbid band along the mucosal surface. It has been proposed that the MTB may represent an earlier stage of intestinal metaplasia, whereas the LBC tends to emerge with more advanced or established GIM. However, the consistency and reproducibility of this proposed progression remain uncertain.

### 4.5. Advanced Imaging for the Detection of Atrophic Gastritis and Gastric Intestinal Metaplasia

Most studies have shown improved detection of gastric IM with enhanced imaging when compared to WLE. Narrow-band imaging (NBI) remains the most extensively studied image-enhanced endoscopy technique for gastric precancerous conditions. Although GIM is relatively straightforward to distinguish from the normal mucosa in the corpus, additional characteristics are required to identify GIM in the antrum, which are more easily detected with enhanced imaging. For example, the light blue crest (LBC) and the marginal turbid band are best seen with NBI and magnification [39,40]. The light blue crest (LBC) demonstrates moderate interobserver reliability, with a kappa coefficient of approximately 0.49, but is highly specific for gastric intestinal metaplasia (GIM), with a specificity reported at around 96%. In a study by Kanemitsu et al., the diagnostic performance of the LBC for histologically confirmed intestinal metaplasia showed a sensitivity of 62.5% and a specificity of 93.8% [41]. The presence of a white opaque substance, corresponding to intraepithelial lipid droplets obscuring the subepithelial capillary network, demonstrated a lower sensitivity of 50.0% but an extremely high specificity of 100.0% (95% confidence interval 85.0% to 100.0%). Importantly, when the LBC and white opaque substance are assessed in combination, the overall sensitivity improves substantially, reaching approximately 87% while maintaining a high specificity of around 93.8%. These and additional studies have been tabulated in the British Society guidelines [11].

A recent systematic review and meta-analysis demonstrated that narrow-band imaging (NBI) provides strong diagnostic performance for the detection of gastric intestinal metaplasia (GIM). On a per-patient basis, the pooled sensitivity was approximately 0.79 (95% confidence interval 0.72–0.85) and the specificity was 0.91 (95% confidence interval 0.88–0.94). Diagnostic accuracy was further improved when assessed on a per-biopsy basis, with sensitivity increasing to 0.84 and specificity to 0.95. Among the evaluated endoscopic features, the tubulovillous mucosal pattern emerged as the most accurate marker for identifying GIM. Notably, this pattern can be reliably recognised even without the need for high magnification, enhancing its practical utility in routine endoscopic assessment [42].

The Endoscopic Grading of Gastric Intestinal Metaplasia (EGGIM) score represents an alternative endoscopy-based staging system for assessing the extent of gastric intestinal metaplasia (IM). This approach utilises narrow-band imaging (NBI) to systematically evaluate five predefined anatomical regions of the stomach, with each area scored according to the estimated proportion of mucosal involvement (less than or greater than a defined threshold of surface involvement). Targeted biopsies are subsequently obtained to provide histological confirmation of the endoscopic findings. Validation studies comparing the EGGIM score with the established histological OLGIM staging system have demonstrated good concordance, suggesting that this endoscopic staging strategy may offer a clinically effective and practical tool for risk stratification and management of patients with gastric intestinal metaplasia [43].

Linked colour imaging (LCI) is a more recent image-enhanced endoscopic modality that combines narrow-band wavelength illumination with conventional white-light imaging to accentuate mucosal colour contrast and improve lesion visibility. Randomised controlled trials have demonstrated that LCI achieves higher detection rates than standard white-light imaging for atrophic gastritis, highlighting its enhanced sensitivity for subtle mucosal changes (2.19% vs. 0.55%) [44]. A subsequent meta-analysis in 2024 involving 7836 patients, incorporating multiple studies and a large patient cohort, further confirmed that LCI significantly improves the detection of both early gastric cancer and gastric intestinal metaplasia (GIM) when compared with white-light imaging. In particular, the detection rate for GIM was markedly higher with LCI, indicating a substantial diagnostic advantage (detection rate of gastric intestinal metaplasia: 88.9% vs. 40.1%, *p* = 0.0003) [45]. On LCI, areas of intestinal metaplasia typically appear as a distinctive lavender or purple discoloration of the mucosa. A characteristic feature, termed the “purple in mist” sign, has been shown to have high diagnostic accuracy, with a reported sensitivity approaching 90% and a specificity exceeding 90% for the identification of intestinal metaplasia [46,47].

Introduced in 2020, texture and colour enhancement imaging (TXI), represents one of the most recent developments in image-enhanced endoscopy. TXI is designed to improve visualisation by enhancing overall brightness, accentuating subtle surface irregularities, and increasing the contrast of minor colour differences within the mucosa [48]. Clinical studies have demonstrated that TXI significantly improves the visibility of mucosal atrophy compared with conventional white light imaging, reflected by higher visibility scores and clearer delineation of atrophic changes (visibility score: 3.8 ± 0.5 vs. 2.8 ± 0.9) [48]. In addition, randomised controlled trial data have shown that both TXI and narrow-band imaging (NBI) significantly enhance colour contrast between abnormal and surrounding mucosa, particularly in areas of atrophy and gastric intestinal metaplasia, when compared with white-light imaging (atrophy: TXI vs. WLI *p* = 0.003, NBI vs. WLI) [49]. Notably, while both modalities improve visualisation, NBI has been shown to detect intestinal metaplasia at a significantly higher rate than white-light imaging, underscoring its continued importance in the endoscopic assessment of premalignant gastric conditions.

Optical enhancement with magnification endoscopy (ME-OE) has demonstrated promising diagnostic performance in clinical studies. Evidence from a randomised controlled trial showed that ME-OE achieved a higher per-patient diagnostic yield for gastric intestinal metaplasia (GIM), gastric intraepithelial neoplasia, and early gastric cancer when compared with conventional white-light endoscopy. (36.6% vs. 23.8%, *p* = 0.018) [50]. When evaluated specifically for gastric intestinal metaplasia, ME-OE compared to WLE demonstrated significantly superior diagnostic performance, including higher sensitivity (82.8% vs. 54.3%, *p* = 0.003), positive predictive value (88.9% vs. 70.4%, *p* = 0.038), and accuracy (83.3% vs. 69.2%, *p* = 0.028) sensitivity, positive predictive value, and overall accuracy [50]. These findings highlight the added value of magnification combined with optical enhancement, enabling improved visualisation of microstructural and microvascular patterns, and thereby enhancing the detection and characterisation of premalignant and early neoplastic gastric lesions.

### 4.6. Advanced Imaging for Gastric Neoplasia Detection

Assessment of the high-risk stomach should always be performed using high-definition white-light endoscopy. Endoscopic findings that raise concern for neoplasia include the distortion of glandular architecture and irregular microvascular patterns, often associated with the disruption or loss of the normal pit pattern and mucosal surface structure. In addition, the presence of a persistent or non-healing gastric ulcer should prompt suspicion for underlying neoplastic pathology.

Combining white-light endoscopy with narrow-band imaging (NBI) and magnification improves the detection of early gastric cancer (EGC). This approach is particularly effective for identifying depressed-type EGC, where it demonstrates a very high sensitivity of 95% and a specificity of 96.8% for depressed EGCs, supporting its role in the detection of subtle early lesions [51].

The Japanese Vascular Surface (VS) classification, first described in 2009, provides a systematic method for lesion characterisation through the independent evaluation of the microvascular (MV) pattern and microsurface (MS) patterns. Within this framework, key features suggestive of EGC include the identification of a distinct demarcation line, with irregular MV and MS patterns present inside this boundary [52].

Further subclassification of the microvascular pattern includes:

- a fine network pattern, characterised by a mesh-like vascular configuration;
- a corkscrew pattern, consisting of tortuous, irregular vessels lacking clear interconnections.

Studies assessing the performance of magnification NBI using the VS classification system have demonstrated high diagnostic accuracy, with sensitivity and specificity reaching very high levels of 95% and 96% respectively for the detection of early gastric cancer [53].

In a systematic review and meta-analysis, NBI showed a pooled sensitivity and specificity of 0.87 and 0.97 on a per-biopsy basis for dysplasia [42]. Magnifying NBI with the vessel plus surface (VS) classification improved performance for characterising early gastric cancer.

Endocytoscopy (EC) is an advanced imaging technique that enables ultra-high magnification, allowing direct visualisation of the mucosa at near-cellular resolution in vivo. It has been explored as a method to improve the detection of early superficial gastric lesions, including signet-ring cell carcinoma and gastric lymphoma. Despite its potential, EC currently lacks a standardised classification system for gastric lesion assessment, and its availability remains largely limited to specialised centres and research environments, restricting its routine clinical use.

## 5. Artificial Intelligence Applications

### 5.1. Diagnostic Performance of AI Systems

Artificial intelligence (AI) is increasingly being recognised as a valuable adjunct to enhanced endoscopic imaging in the detection of gastric precancerous conditions. In a multicentre diagnostic study evaluating the ENDOANGEL system, the AI platform demonstrated high diagnostic accuracy, achieving 90.1% for gastric atrophy and 90.8% for intestinal metaplasia in internal test datasets, with comparable results observed during prospective video-based assessment [54]. When benchmarked against human performance, the AI system achieved diagnostic accuracy comparable to expert endoscopists (86.9% versus 84.6% for gastric atrophy), with no statistically significant difference. Notably, its performance was significantly superior to that of non-expert endoscopists, who demonstrated lower diagnostic accuracy (75.0%, *p* = 0.008). These findings highlight the potential role of AI as a supportive tool to enhance diagnostic consistency and accuracy in the endoscopic evaluation of premalignant gastric pathology.

Individual deep learning models have demonstrated impressive performance. The GAM-EfficientNet model achieved a sensitivity of 93%, a specificity of 94%, and an accuracy of 93.5% in external testing, with video test performance showing 96.23% sensitivity, 89.23% specificity, and 92.37% accuracy, all exceeding the performance of three endoscopists [55].

Furthermore, a systematic review and meta-analysis reported that AI-based systems achieve a pooled diagnostic accuracy of over 90% for the detection of gastric precancerous lesions, underscoring their potential role in improving diagnostic precision and consistency [56]. A similar systematic review and meta-analysis demonstrated excellent diagnostic accuracy for detecting chronic atrophic gastritis and gastric intestinal metaplasia, with pooled sensitivity of 94% and specificity of 96% for atrophic gastritis detection [57].

### 5.2. Evidence from Randomised Controlled Trials

The largest multicenter randomised controlled trial (29,514 patients from 24 hospitals) found that AI did not significantly improve the detection rate of gastric neoplasms after pathological review (1.42% vs. 1.25%, RR 1.13, 95% CI 0.92–1.38, *p* = 0.25) [58]. However, based on the original pathology before expert review, AI showed a marginal improvement (4.06% vs. 3.57%, RR 1.14, 95% CI 1.0–1.28, *p* = 0.03). Importantly, AI reduced blind spots from 2.52 to 1.07.

A 2026 meta-analysis of 11 randomised controlled trials involving 57,512 participants found that AI-assisted endoscopy demonstrated higher detection rates per patient (RR 1.57, 95% CI 1.23–2.01) and per lesion (RR 1.55, 95% CI 1.33–2.18) [59]. The benefit was more evident for lesions ≤10 mm (RR 2.24, 95% CI 1.72–2.91), with higher detection rates across all histological subtypes including low-grade intraepithelial neoplasia (RR 1.73, 95% CI 1.30–2.32) [59].

A single-centre tandem randomised trial demonstrated that AI-assisted endoscopy significantly reduced the gastric neoplasm miss rate from 27.3% to 6.1% (relative risk 0.224, 95% CI 0.068–0.744, *p* = 0.015) [60]. This represents a 78% reduction in missed gastric neoplasms when AI was used first compared to routine endoscopy.

### 5.3. AI for Risk Stratification and Staging

Deep learning models employing semi-supervised learning have demonstrated promising diagnostic performance in the assessment and grading of intestinal metaplasia and gastric atrophy, achieving area under the curve (AUC) values of 0.884 and 0.877, respectively, in external validation datasets [61]. The integration of artificial intelligence support has also been shown to enhance pathologist performance, with significant improvements observed in the AUC, sensitivity, and interobserver agreement (weighted kappa) when compared to unaided assessment in the diagnosis of intestinal metaplasia [61].

Emerging evidence from a recent study conducted in 2026 underscores the potential role of artificial intelligence (AI) in addressing inter-observer variability in the quantification of intestinal metaplasia, which remains a key limitation of conventional visual estimation methods [62]. AI-based models generate more consistent and standardised assessments, offering a means to reduce subjectivity in risk stratification. However, agreement between pathologists and AI systems is currently only moderate, with reported kappa values ranging from 0.12 to 0.35. Therefore, visual estimation remains the only available method, despite the considerable inter-observer variability. Deep learning models provide consistent estimates that could help reduce this subjectivity in risk stratification and thus may provide a supportive role.

### 5.4. Current Limitations and Future Directions in AI

Despite promising results, several challenges remain. A 2026 review on gastric intestinal metaplasia diagnosis notes that challenges include the need for external validation, the management of data heterogeneity, the generalizability of models beyond Asian cohorts, and the development of explainable AI [63]. Generalisation also remains a significant concern. Many AI models are trained using datasets from single-centre or geographically limited populations, often in East Asia where gastric cancer prevalence is high and endoscopic expertise is well developed. Consequently, algorithm performance may not translate consistently across Western populations, different ethnic groups, or centres using alternative imaging systems. External validation studies remain relatively limited, and concerns persist regarding overfitting and reproducibility.

Interobserver variability in histopathologic diagnosis represents a significant challenge. A 2026 study found that among pathologists, inter-observer agreement by Fleiss’s Kappa score ranged from only 0.20 to 0.48 for intestinal metaplasia assessment, while agreement between pathologists and AI ranged from 0.12 to 0.35 [62]. This highlights that AI models may provide more consistent estimates than visual estimation, which is marked by considerable inter-observer variability.

Although gastric intestinal metaplasia and dysplasia are endoscopically detectable, findings often go undiagnosed when endoscopists are unfamiliar with their characteristic visual features. There is an unmet need for improved training, especially in the West, where AI tools could potentially address this gap.

Despite major advances in artificial intelligence (AI)-assisted endoscopy, substantial barriers remain before these systems can be fully integrated into routine clinical practice. One of the principal challenges is the high financial cost associated with their implementation. AI-enabled endoscopy requires advanced processor platforms, high-definition imaging systems, compatible software licences, data storage infrastructure, and ongoing technical support. These costs may be manageable in tertiary academic centres but are often prohibitive for smaller hospitals and healthcare systems with limited resources, potentially widening disparities in access to high-quality diagnostic care.

Another important limitation relates to variability in real-time processing performance. AI systems rely on rapid image acquisition and analysis to provide accurate lesion detection during live endoscopy. However, processing speed can be affected by hardware limitations, image quality, motion artefacts, poor mucosal preparation, and differences between endoscopy platforms. Even small delays in image interpretation may reduce the practical usefulness of AI during dynamic procedures, particularly when assessing subtle lesions such as early gastric cancer or intestinal metaplasia. Furthermore, many AI algorithms demonstrate excellent performance under controlled research conditions but may perform less reliably in routine clinical environments with variable operators and patient populations.

Medicolegal responsibility represents another unresolved issue. At present, AI functions as a decision-support tool rather than an autonomous diagnostic system, meaning that ultimate clinical responsibility remains with the endoscopist. However, this creates complex legal and ethical questions. If an AI system fails to detect a lesion, responsibility may still fall upon the physician despite their reliance on approved software. Conversely, clinicians may face liability if they disregard AI-generated alerts that subsequently prove correct. The absence of clear legal frameworks defining accountability between clinicians, hospitals, and AI manufacturers remains a major obstacle to widespread adoption.

There are also concerns regarding clinician overreliance on AI systems. Excessive dependence may reduce vigilance and potentially erode diagnostic skills over time, particularly among less experienced endoscopists. AI systems may also increase false-positive detections, leading to unnecessary biopsies, prolonged procedure times, increased costs, and patient anxiety.

Data governance and patient privacy introduce additional challenges. AI development requires access to very large endoscopic image databases, raising important questions regarding consent, anonymisation, cybersecurity, and the ownership of clinical data. Regulatory oversight remains heterogeneous internationally, with evolving standards regarding validation, transparency, and algorithm updates.

Finally, the successful integration of AI into endoscopy will require a substantial investment in structured training and workflow redesign. Endoscopists must understand both the capabilities and limitations of AI systems in order to use them safely and effectively. Future progress will depend not only on technological improvements but also on robust clinical validation, clear regulatory frameworks, equitable accessibility, and careful integration into real-world clinical practice.

Future research priorities include prospective multicenter validation studies, real-time video-based AI systems, the integration of explainable AI for clinical trust, the assessment of their impact on patient outcomes (mortality and interval cancer incidence), and evaluation across varying levels of endoscopist experience [63,64]. The technology shows particular promise for assisting less experienced endoscopists and in resource-limited settings where expertise may be limited [58,65].

The 2025 AGA Clinical Practice Update stated that although AI technologies show promise in the detection of early gastric neoplasia, the current evidence base remains insufficient to support routine clinical implementation [66].

## 6. Conclusions

Gastric cancer remains a significant worldwide issue. Although the overall age-standardised incidence is declining, the absolute number of cases is increasing due to a paradoxical increase in individuals under 50 and an ageing population. The prognosis is improved by the treatment of early disease. At present the most effective strategy for early detection is endoscopy, in particular targeting those at the highest risk with CAG. The introduction of advanced imaging has greatly improved the ability to effectively survey those at risk and detect dysplasia and early cancer. However, training and awareness remain sub-optimal, with low diagnosis rates of CAAG and high PEUGIC rates during surveillance. AI has shown promise in improving the detection and staging of CAG, as well as the detection of early gastric cancer. However, multicenter prospective validation studies are required before AI is incorporated into standard practice and guidance.

## Data Availability

The original contributions presented in this study are included in the article. Further inquiries can be directed to the corresponding author.
